# Prevalence and antimicrobial resistance of *Campylobacter jejuni* and *Campylobacter coli* isolated from children and environmental sources in urban and suburban areas

**DOI:** 10.1186/s12866-017-0991-9

**Published:** 2017-04-04

**Authors:** Bernadeta Szczepanska, Małgorzata Andrzejewska, Dorota Spica, Jacek J. Klawe

**Affiliations:** Department of Hygiene, Epidemiology and Ergonomics, Nicolaus Copernicus University in Torun, Collegium Medicum in Bydgoszcz, 9 Sklodowska-Curie Str, 85-094 Bydgoszcz, PL Poland

**Keywords:** *Campylobacter*, Children, Pets, Poultry meat, Surface water, Fountains, Antimicrobial resistance

## Abstract

**Background:**

Campylobacteriosis is a dominant bacterial cause of foodborne infection and is considered the main public health problem in Europe and many other countries worldwide. In the study lasting from 2011 to 2013 we compared the prevalence and antimicrobial resistance of *Campylobacter jejuni* and *Campylobacter coli* isolated from children, domestic animals, poultry meat and surface water in Northern Poland.

**Results:**

During a 3-years study 1973 samples were analysed. The results proved the presence of *Campylobacter* spp. in 306 (15.5%) samples. The percentage of *Campylobacter*-positive samples differed among the sample types, from 0% (freshwater beaches) to 38.6% (poultry meat in 2011). Prevalence of *Campylobacter* spp. in children isolates was 9.6%. It decreased from 13.2% in 2011 to 8.0% in 2013. It should be highlighted with a particular concern that *Campylobacter jejuni* was detected in 20.0% of fountains. All children and poultry meat isolates were susceptible to azithromycin. Two *C. coli* (3.7%) and four *C. jejuni* (3.3%) isolated from poultry meat were resistant to erythromycin. The highest percentage of *C. jejuni* isolates with resistance to ciprofloxacin were found in samples from 80% dogs and 85% ponds. Among isolates resistant to two antimicrobials 74.7% *C. jejuni* and 59.2% *C. coli* isolates were resistant to ciprofloxacin as well as to tetracycline. Only one cat *C. coli* isolate was resistant to both azithromycin and erythromycin. One *C. jejuni* isolate from a fountain was resistant to four antimicrobial agents (erythromycin, azithromycin, tetracycline and ciprofloxacin).

**Conclusions:**

The study proved that surface water, poultry meat and pets constituted potential sources of *Campylobacter* to children. Fountains can be a direct source of children campylobacteriosis but can also pollute other environments with multidrug-resistant *Campylobacter*. The high resistance to some antimicrobials among the isolates may lead to increasing numbers of difficult-to-treat campylobacteriosis cases among children.

## Background

Campylobacteriosis is a dominant bacterial cause of foodborne infection and is considered the main public health problem in Europe and many other countries worldwide [1, 2]. The natural reservoirs of *Campylobacter* spp. are intestinal tracts of domesticated and wild birds and mammals. Eating or handling with raw or undercooked meat, especially poultry, is considered to be major risk factors for human campylobacteriosis. Other sources of *Campylobacter* are the following: contaminated drinking water and dairy products, for example unpasteurised milk, swimming in natural water sources and contact with pets [2–4]. Foreign travel may also be a risk factor for *Campylobacter* infection, especially in small children previously unexposed to exotic or antibiotic-resistant strains present in contaminated meat or water [5, 6].

In humans, *Campylobacter jejuni* and *Campylobacter coli* are pathogens, routinely causing acute diarrhoea, but sometimes Guillain-Barré syndrome, reactive arthritis and postinfectious polyneuropathy leading to paralysis may occur [7, 8].

As a generally self-limiting disease *Campylobacter* infection does not require therapeutic intervention. In children with fever, increasing bloody diarrhoea or symptoms lasting longer than 1 week and in those who are immunologically compromised antimicrobial treatment should be considered. The drugs of choice used in the clinical therapy of campylobacteriosis are macrolides, such as azithromycin and erythromycin, and fluoroquinolones, such as ciprofloxacin; the first ones are considered to be safe therapeutic agents for children, while the second ones are used occasionally in paediatric patients. Tetracyclines could be considered as an alternative choice in the therapy of *Campylobacter* infection, but in practice they are not often used [7, 9–11]. According to the results of numerous studies a significant rise in resistance to fluoroquinolones, tetracycline, and erythromycin has been demonstrated in *C.jejuni* and *C. coli* isolates from various sources such as humans, animals and food [12–15]. What should be stressed is that the number of studies with regard to isolates from surface water is limited.

Studies in many countries have shown that natural environment, like soil and water, is essential in transmission of *Campylobacter*, either directly to humans or indirectly through farm animals or pets [5, 6, 16]. One of the pathways for spreading of *Campylobacter* are faeces of wild and domestic animals present in recreational and drinking water. Children can be in contact with animal excrement in the environment during outdoor activities on children’s playgrounds or in parks.

The subject of the study was to compare the prevalence and antimicrobial resistance of *C. jejuni* and *C. coli* isolated from children, domestic animals, poultry meat and surface water in urban and suburban areas of the Bydgoszcz region in Poland. The role of environmental exposures in the epidemiology of *Campylobacter* infection in children under 4 years of age was also analysed.

## Methods

### Sample collection

Stool samples from 1030 children aged 0–4 with diarrhoea were obtained from the Infectious Diseases Hospital in Bydgoszcz, Northern Poland. We examined 433 samples of poultry meat from randomly selected supermarkets and butcher shops in the study region. Among the said samples there were chicken filets, chicken wings, chicken leg quarters, chicken drumsticks, turkey filets. Rectal swabs were collected from 260 pets (146 dogs and 114 cats). The samples were taken from young animals aged from 2 weeks to 24 months, living with their owners, in shelters, as well as from veterinary clinics during routine check-ups. The animals had no signs of gastrointestinal disease. A total of 250 surface water samples (from ponds and ornamental lakes, freshwater beaches, Brda and Vistula rivers and city fountains) were examined in the study. Table 1 presents all samples collected and tested over a period of 3 years from 2011 to 2013.

**Table 1:** Prevalence of *Campylobacter* isolated from different sources in Poland from 2011 to 2013

Sample type	Year/sample type	No. of samples tested	No. (%) of samples positive for *Campylobacter*	No. (%) of samples positive for	
				*C. jejuni*	*C. coli*
Children	2011	264	35 (13.3)	32 (12.1)	3 (1.1)
	2012	393	34 (8.7)	32 (8.1)	2 (0.5)
	2013	373	30 (8.0)	28 (7.5)	2 (0.5)
	Total	1030	99 (9.6)	92 (8.9)	7 (0.7)
Poultry meat	2011	140	54 (38.6)	29 (20.7)	25 (17.9)
	2012	143	42 (29.4)	31 (21.7)	11 (7.7)
	2013	150	48 (32.0)	30 (20.0)	18 (12.0)
	Total	433	144 (33.3)	90 (20.8)	54 (12.5)
Pets	dogs	146	9 (6.2)	5 (3.4)	4 (2.7)
	cats	114	12 (10.5)	10 (8.8)	2 (1.8)
	Total	260	21 (8.1)	15 (5.8)	6 (2.3)
Surface water	Rivers	40	7 (17.5)	5 (12.5)	2 (5.0)
	Ponds and ornamental lakes	150	30 (20.0)	20 (13.3)	10 (6.7)
	Freshwater beaches	35	0	0	0
	Fountains	25	5 (20.0)	5 (20.0)	0
	Total	250	42 (16.8)	30 (12.0)	12 (4.8)
Total		1973	306 (15.5)	227 (11.5)	79 (4.0)

### *Isolation of Campylobacter* spp.

Isolation of *Campylobacter* spp. from stool samples was performed according to the World Health Organization recommendations [17], with the use of Charcoal Cefoperazone Deoxycholate Agar (CCDA) (Oxoid, Basingstoke, United Kingdom). Plates were incubated at 42 °C for 48 h in a microaerobic atmosphere generated by a Generbox microaer (BioMerieux, Marcy l’Etoile, France). Isolation of *Campylobacter* spp. from poultry meat was conducted in compliance with the EN ISO 10272–1:2006 method [18]. Twenty five grams of meat were placed into 225 ml of Bolton broth (Oxoid) containing the Bolton broth selective supplement (Oxoid) and 5% laked horse blood (Oxoid). Next, bacterial suspension was spread onto CCDA plates, and then incubated at 42 °C for 48 h under microaerobic conditions. Isolation from water samples was performed in accordance with the recommendations of the Health Protection Agency [19]. Water samples were filtered through a 0.45 μm filter (Millipore 0.45) and the filter was then transferred to 100 ml of Bolton broth containing the Bolton broth selective supplement (Oxoid). The broths were incubated at 42 °C in a microaerobic atmosphere for 48 h and then streaked on CCDA plates. Rectal swabs from domestic animals were stored at 4 °C in a transport medium Amies Agar Gel - With Charcoal (Copan Italia, Brescia, Italy), transmitted onto the Bolton broth containing the Bolton broth selective supplement (Oxoid), and then incubated under the conditions described above. Broth cultures were streaked onto CCDA plates and incubated at 42 °C under microaerobic conditions for 48 h. Characteristic growth from CCDA plates for all types of samples was placed on a blood plate (Columbia agar with 5% sheep blood) (Oxoid) and incubated overnight at 42 °C. Suspected colonies were confirmed as *Campylobacter* on the base of cell morphology by the Gram staining method and motility. An oxidase test was performed using Oxidase Identification Sticks (Oxoid) in compliance with the manufacturer’s instructions. The hippurate hydrolysis test with a 3.5% ninhydrin solution was used to identify *C. jejuni* isolates [17]. Subsequently, two suspected colonies per sample were selected and streaked on Columbia agar containing 5% sheep blood (Oxoid) and incubated at 37 °C for 48 h under microaerobic conditions. All confirmed and purified *Campylobacter* isolates were stored in the archive at -80 °C in Microbanks (Pro-Lab Diagnostics, United Kingdom).

### DNA extraction

Genomic DNAs were obtained from 24-h cultures on Columbia agar containing 5% sheep blood by a conventional boiling method [20]. Bacterial colonies were suspended in 100 μl PBS with 45 μl Chelex 100 chelating resin (BioRad, USA) and next boiled for 10 min. The samples were cooled on ice and centrifuged at 13,000 x *g* for 10 min. The supernatant was stored at −20 °C. Aliquots of 1 μl of template DNA were used for PCR.

### Identification of Campylobacter spp.

The PCR method with specific primers as described by On and Jordan was used for the purpose of identification of colonies as C. *jejuni* or C. *coli* [21, 22]. The primers for *C.jejuni* were *C. jejuni* Random (5′-CA TCT TCC CTA GTC AAG CCT-3′) [22], resulting in an amplicon of 773 bp, and the primers for *C. coli* were *C. coli* Random (5′-AG GCA AGG GAG CCT TTA ATC-3′) [22], resulting in an amplicon of 364 bp. PCR reactions were performed in final mixture volume of 25 μl containing 1.0 μl of each PCR primer (10 μM) (Oligo, Warsaw, Poland), 2.5 μl of 10× PCR buffer (Fermentas, Vilnius, Lithuania), 1.0 μl of 10 mM deoxynucleoside triphosphate mix (Fermentas), 2.5 μl of 25 mM MgCl_2_ (Fermentas), 0.5 μl of Dream Taq DNA Polymerase (1 U/μl) (Fermentas), 1.0 μl of template and 13.0 μl of DNA-free purified water (Fermentas) [23]. DNA amplification was carried by an initial denaturation step at 94 °C for 5 min, then 30 cycles of 94 °C for 30 s, 61 °C for 30 s and 72 °C for 30 s in a Bio-Rad thermocycler. The final extension was performed at 72 °C for 5 min. The amplified DNAs were analysed by electrophoresis in a 1.5% agarose gel. Gels were visualised by staining them with the Midori Green Stain (Nippon Genetics, Duren, Germany) and photographed by using the IG/L-E InGenius L documentation system (Syngene, Cambridge, United Kingdom). A 100 bp DNA marker (Fermentas) was used as a size marker for the PCR amplicons.

### Antimicrobial susceptibility testing

The susceptibility of *C. jejuni* and *C. coli* to antibiotics was determined with the E-test (AB Biodisk, Solna, Sweden) on Mueller-Hinton agar with 5% defibrinated horse blood (bioMerieux, Marcy l’Etoile, France) and in compliance with the manufacturer’s instructions [24]. Azithromycin, erythromycin, gentamicin, ciprofloxacin and tetracycline - five antimicrobials clinically used and most often tested in both food/animal and human isolates were analysed. The plates were incubated at 37 °C for 48 h under microaerobic conditions. The breakpoints for *Campylobacter* resistance were interpreted by means of criteria for the *Enterobacteriaceae* family according to Clinical and Laboratory Standards Institute [25]: azithromycin, 8 μg/mL; erythromycin, 32 μg/mL; gentamicin, 8 μg/mL tetracycline, 16 μg/mL and ciprofloxacin, 4 μg/mL. *C. jejuni* ATCC 33560, *C. jejuni* ATCC 33291 and *C.coli* ATCC 33559 were used as control strains.

### Statistical analysis

Statistical calculations were performed by means of the Statistica 10.0 program (StatSoft Poland, 2011). The association between the prevalence of *Campylobacter* spp. in individual sample types and antimicrobial resistance between *C. jejuni* and *C. coli* were analysed with a two-proportion test. *P* values of <0.05 were considered statistically significant.

## Results

### Prevalence of Campylobacter spp. in various sources

During our 3-years study 1973 samples from children, domestic animals, poultry meat and surface water were analysed (Table 1). The results have indicated the presence of *Campylobacter* spp. in 306 (15.5%) of the samples. Frequency of *C. jejuni* in the examined samples was 11.5%. *C. coli* was found in 4.0% of the analysed samples. The two-proportion test revealed that *C. jejuni* isolates were significantly more frequently detected than *C. coli* isolates in all types of the examined samples (*p* < 0.5). The samples from poultry meat had the highest prevalence of *Campylobacter* (33.3% in 3 years). The proportion of *Campylobacter*-positive samples varied among various sample types, from 0% (freshwater beaches) to 38.6% (poultry meat in 2011). The prevalence of *Campylobacter* spp. in children isolates was 9.6%. It decreased from 13.2% in 2011 to 8.0% in 2013 (*p* = 0.0323). *Campylobacter* spp*.* was detected in 20.0% of fountains and in all cases it was identified as *C. jejuni*. Over the course of our research, the lowest prevalence of the examined bacteria was observed in 8.1% pets, in particular dogs (6.2%). Statistical hypotheses were tested between two sources. Sources were taken in various combinations into each hypothesis. From this point of view there were statistically significant differences between the prevalence of *Campylobacter* in various sources, with the exception of the prevalence in isolates from children and pets (children vs. pets, *p* > 0.05).

### Antimicrobial resistance

The lowest antimicrobial resistance rate were noted for gentamicin and azithromycin (Table 2). All isolates from children and poultry meat were susceptible to azithromycin. One *C. jejuni* and one *C. coli* isolates from cats, and three *C. jejuni* isolates from surface water (from ponds and fountains) were resistant to azithromycin. Resistance to erythromycin was detected in two *C. coli* (3.7%) and four *C. jejuni* (3.3%) isolated from poultry meat. High rates of ciprofloxacin resistance (>50%) were noted in all isolates. In particular higher level of resistance to fluoroquinolones was found within the *C. coli* isolated from children and from poultry meat (71.4 and 74.1%, respectively) rather than within *C. jejuni* (65.2 and 62.2%, respectively); however, this difference was not statistically significant. The highest percentage of *C. jejuni* isolates resistant to ciprofloxacin was found in samples obtained from 4/5 dogs (80%) and 17/20 ponds (85%). The lower level of resistance was observed in relation to tetracycline.

**Table 2:** *Campylobacter* isolates susceptibility to antibiotics^a^

Antimicrobial agent	*Campylobacter* species	No. (%) of resistant isolates								
		Children	Poultry meat	Domestic animals			Surface water			
				Total	Dogs	Cats	Total	Rivers	Ponds and ornamental lakes	Fountains
Erythromycin	*C. coli*	0/7	2/54 (3.7)	1/6 (16.7)	0/4	1/2 (50.0)	0/12	0/2	0/10	0
	*C. jejuni*	0/92	4/90 (4.4)	0/15	0/5	0/10	3/30 (10.0)	0/5	1/20 (5.0)	2/5 (40.0)
Azithromycin	*C. coli*	0/7	0/54	1/6 (16.7)	0/4	1/2 (50.0)	0/12	0/2	0/10	0
	*C. jejuni*	0/92	0/90	1/15 (6.7)	0/5	1/10 (10.0)	3/30 (10.0)	0/5	1/20 (5.0)	2/5 (40.0)
Gentamicin	*C. coli*	0/7	0/54	0/6	0/4	0/2	0/12	0/2	0/10	0
	*C. jejuni*	1/92 (1.1)	1/90 (1.0)	0/15	0/5	0/10	0/30	0/5	0/20	0/5
Tetracycline	*C. coli*	3/7 (42.9)	17/54 (31.5)	3/6 (50.0)	2/4 (50.0)	1/2 (50.0)	6/12 (50.0)	1/2 (50.0)	5/10 (50.0)	0
	*C. jejuni*	36/92 (39.1)	46/90 (51.1)	5/15 (33.3)	2/5 (40.0)	3/10 (30.0)	18/30 (60.0)	2/5 (40.0)	13/20 (65.0)	3/5 (60.0)
Ciprofloxacin	*C. coli*	5/7 (71.4)	40/54 (74.1)	3/6 (50.0)	2/4 (50.0)	1/2 (50.0)	8/12 (66.7)	1/2 (50.0)	7/10 (70.0)	0
	*C. jejuni*	60/92 (65.2)	56/90 (62.2)	9/15 (60.0)	4/5 (80.0)	5/10 (50.0)	24/30 (80.0)	3/5 (60.0)	17/20 (85.0)	4/5 (80.0)

^a^Results expressed as the number of resistant isolates vs. total number of isolates analyzed

Table 3 shows antimicrobial resistance phenotype patterns among tested *Campylobacter*. Resistance to two or more antimicrobials was found in 40% of *C. jejuni* and 29% of *C. coli* isolates in all types of samples. Among isolates resistant to two antimicrobials 74.7% *C.jejuni* and 59.2% *C. coli* isolates combined resistance to ciprofloxacin and tetracycline. In comparison to C. coli isolates, *C. jejuni* isolates were more often resistant to three or more antimicrobials (2.5% vs. 5.7%). Combination of ciprofloxacin, tetracycline and erythromycin resistance, found in *C. jejuni* isolates from poultry meat and water was observed most frequently within the group resistant to three or more antibiotics. Only one *C. coli* isolate from cats was resistant to both erythromycin and azithromycin. The study revealed that one *C.jejuni* isolate from a fountain was resistant to four antimicrobial agents (erythromycin, azithromycin, tetracycline and ciprofloxacin).

**Table 3:** Antimicrobial resistance phenotype patterns among the tested *Campylobacter* (results expressed as the number of resistant isolates vs. total number of isolates analyzed)

Antimicrobial resistance phenotype	*Campylobacter species*	No. (%) of resistant isolates			
		Children	Poultry meat	Pets	Surface water
Sensitive for all	*C. coli*	1/7 (14.3)	8/54 (14.8)	2/6 (33.3)	2/12 (16.7)
	*C. jejuni*	14/92 (15.2)	10/90 (11.1)	4/15 (26.7)	5/30 (16.7)
AZM + EM	*C. coli*	0/7	0/54	1/6 (16.7)	0/12
	*C. jejuni*	0/92	0/90	0/15	2/30 (6.7)
TC + CI	*C. coli*	2/7 (28.6)	10/54 (18.5)	2/6 (33.3)	2/12 (16.7)
	*C. jejuni*	34/92 (37.0)	12/90 (13.3)	4/15 (26.7)	9/30 (30.0)
TC + EM	*C. coli*	0/7	0/54	0/6	0/12
	*C. jejuni*	0/92	2/90 (2.2)	0/15	3/30 (10.0)
TC + AZM	*C. coli*	0/7	0/54	0/6	0/12
	*C. jejuni*	0/92	0/90	0/15	3/30 (10.0)
CI + EM	*C. coli*	0/7	2/54 (3.7)	1/6 (16.7)	0/12
	*C. jejuni*	0/92	3/90 (3.3)	0/15 (0)	3/30 (10.0)
CI + AZM	*C. coli*	0/7	0/54	1/6 (16.7)	0/12
	*C. jejuni*	0/92	0/90	1/15 (6.7)	3/30 (10.0)
CI + AZM + EM	*C. coli*	0/7	0/54	1/6 (16.7)	0/12
	*C. jejuni*	0/92	0/90	0/15	2/30 (6.7)
CI + TC + AZM	*C. coli*	0/7	0/54	0/6	0/12
	*C. jejuni*	0/92	0/90	0/15	3/30 (10.0)
CI + TC + EM	*C. coli*	0/7	1/54 (1.9)	0/6	0/12
	*C. jejuni*	0/92	2/90 (2.2)	0/15	3/30 (10.0)
TC + AZM + EM	*C. coli*	0/7	0/54	0/6	0/12
	*C. jejuni*	0/92	0/90	0/15	2/30 (6.7)
TC + CI + AZM + EM	*C. coli*	0/7	0/54	0/6	0/12
	*C. jejuni*	0/92	0/90	0/15	1/30 (3.3)

*EM* resistance to erythromycin, *AZM* resistance to azithromycin, *CI* resistance to ciprofloxacin, *TC* - resistance to tetracycline

## Discussion

Bydgoszcz is a city in northern Poland, on the Brda and Vistula rivers and the Bydgoszcz Canal, with an urban agglomeration with more than 470,000 inhabitants. About 35% of the city’s area is covered with parks and recreational areas with numerous ponds and fountains. We were interested in the influence of these environment agents on campylobacteriosis in young children (0–4). In several studies, this age group was indicated as a group of high risk of campylobacteriosis [26–28]. It can be a result of not fully established immune response, as well as deficient hand hygiene and contact with animals and the environment. In our study we emphasized specific environmental urban and suburban area agents that could play a role in campylobacteriosis in children. Faeces of birds, stray dogs, cats and other wild animals inhabiting many playgrounds and parks in urban and suburban areas can be the environmental source of campylobacteriosis for children [16, 29]. Due to the fact ornamental lakes and fountains are contaminated with the animals’ faeces they become a potential source of *Campylobacter* to children.

In the study, *Campylobacter* was isolated from 99 (9.6%) samples from children with diarrhoea. *C. jejuni* (8.9%) was isolated more often than *C. coli* (0.7%). Our results are similar to those obtained in Warsaw (Poland) by Rozynek et al. [15]. In England and Wales, as well as in other industrialised countries, *C. jejuni* was present in 5–16% of children with diarrhoea, while frequency of these bacteria in healthy children was from 0 to 1.5% [28, 30, 31].

Handling and consumption of chicken meat were described as the main risk factors for human campylobacteriosis in many of the analytical epidemiological studies [32–34]. Infants and young children could become infected with *Campylobacter* by touching contaminated poultry meat or contaminated equipment. Contaminated hands of caretakers can be also a source of infection [35]. In this study, *Campylobacter* was detected in 33.3% of tested chicken meat samples, thus confirming that the risk of infections is high. In our study conducted in the same area during 2009–2013, the occurrence of *Campylobacter* in retail poultry meat was higher (41.6%) and exhibited a decreasing temporal trend from 60.2% in 2009 to 32.0% in 2013 [23]. The decrease of *Campylobacter* spp. level in poultry carcasses from 70% in 2009 to 39% in 2013 was also observed and presented by Department of Hygiene of Food of Animal Origin National Veterinary Research Institute in Pulawy [36]. This decreasing trend in Poland could be considered to be a result of the implementation of the European Union standards on how to produce poultry meat with a very low contamination level of *Campylobacter*.

Sporadic campylobacteriosis are connected with contact with pets [37–39]. Fullerton et al. [35] suggest that infants older than 6 months are physically closer to pets and their equipment, which increases a risk of transmission. Frequency of isolation of *Campylobacte*r spp. in dogs varied from 17% (Brazil) to 76.2% (Denmark), while in cats isolation values ranged from 8% in Brazil to 47.8% in Germany [40–42]. In our study the prevalence of *Campylobacter* isolates in dogs was that of 6.2% and it was lower than that in other studies as well as 10.5% of the samples obtained from cats tested positive for *Campylobacter* spp. According to TNS Polska (Public Opinion Research Centre), in 2014 in Poland (population: 38.5 million) there were about 8 million dogs and 6 million cats, with 48% of the country’s citizens owning at least one dog or cat [43, 44]. This interaction between pets and children raises concerns regarding potential zoonotic risks.

The prevalence of *Campylobacter* in surface water varies from 0 to 87.5% as reported [45, 46]. Over the course of our 3-years study, contaminated surface water was a potential reservoir of *Campylobacter*; with 16.8% of total and 17.5% of river water samples testing positive for *Campylobacter*. *Campylobacter* has not been detected in freshwater beach samples, thus indicating that the risk of outbreaks during swimming in the Bydgoszcz region is low. By contrast, 20.0% of water samples taken from fountains and ornamental lakes contained *Campylobacter*. These facilities are very popular in public parks and town centres. When it is hot children like to play in the fountain water that may be contaminated by enteric pathogens. Accidental defecation and rinsing of contaminated human bodies increase the level of contamination of recreational water. Fountains often serve as drinking bowls for birds or stray dogs and cats, and may contribute to the transmission of pathogenic species of microorganisms [27]. Outbreaks of norovirus [47], *Shigella sonnei* [48], legionnaires’ disease [49] associated with a recreational spray or fountain water were reported in some countries but hard evidence studies on the role of decorative pools and fountains in *Campylobacter* pathogenesis are still necessary.

A great number of environmental and animal reservoirs makes the epidemiology of *Campylobacter* infection very complicated. A risk factor of campylobacteriosis connected with food [4, 50] and faecal material from livestock [38, 51] has been extensively studied. Urban wild birds such as ducks, goose, starlings [52, 53], as well as birds inhabiting human settlements, such as white stork [54], contribute to enormous faecal contamination of the environment and need to be treated as an important source of campylobacteriosis for humans and farm animals.

The same *Campylobacter* isolates in children stool samples, surface water and poultry were reported in a previous study conducted in the Bydgoszcz region [55]. Our present study showed lower prevalence of *C. coli* in all sources tested (7, 37.5 and 32% of children, chicken, and water isolates, respectively) than in the study during 2006–2008 (42, 75, and 78%, respectively). Despite a common geographic location these studies were different over time. However, it should be noted that places where samples for research were gathered were different as well. In our previous study chicken isolates were obtained in one poultry slaughterhouse located in the rural area and water samples were collected from rivers and ponds in both urban and rural areas. In our study, poultry meat samples were taken from supermarkets and retail butcher shops, which may indicate different sources of origin of the poultry. Environmental water samples were obtained from different sites located within urban and suburban areas and the samples were taken from rivers, ponds, as well as from ornamental lakes and city fountains that had not been tested before. It may prove circulation of *Campylobacter* spp*.* between poultry, humans, surface water and pets in the area. Further studies on this subject with the use of molecular typing methods need to be conducted to ascertain the transmission of *Campylobacter* within the environment.

In recent years antimicrobial resistance in foodborne pathogens, including *Campylobacter*, is one of essential public health problems [7, 13, 56]. Antibiotics used for growth-promotion in rearing of animals are used at low levels but for a long period of time; consequently, the emergency of resistant bacteria is significant. Due to veterinary applications of enrofloxacin and other quinolones the emergency of ciprofloxacin resistance of *Campylobacter* isolated from poultry and human is observed [13, 57]. The research confirmed a high level of ciprofloxacin resistance (>50%) among all *Campylobacter* isolates, especially in *C. jejuni* isolated from 85% ponds and 80% dogs and in *C. coli* isolated from children and poultry meat (71.4 and 74.1%, respectively). High resistance to fluoroquinolones and quinolones make these agents useless in therapy of children and adults *Campylobacter* infections. Reports from Poland [15, 58] and other countries [13, 59, 60] confirm this statement. The high rate of resistance to ciprofloxacin in isolates obtained from children may result from a transmission of animals (including poultry and pets), since quinolones are used only occasionally in paediatric patients, mainly in the treatment of cystic fibrosis. Furthermore, tetracyclines are used in veterinary medicine in Poland, and high resistance to them was observed in *Campylobacter* spp. of poultry, cattle and pigs origin in our country [7, 15, 61]. In the study, the overall tetracycline resistance observed did exceed 30%, and there were no significant differences in tetracycline resistance between all isolates.

In spite of reports from some parts of the world reporting a slowly increasing resistance rate to macrolides in *Campylobacter*, these antibiotics are the optimal drugs for treatment of campylobacteriosis especially in paediatric patients [7, 12, 62]. In this study, all children isolates were found to be susceptible to azithromycin and erythromycin, but resistance to these drugs was detected in some *Campylobacter* isolates from cats, ponds and fountains. Among *Campylobacter* isolated from poultry meat a low rate of resistance to erythromycin in general was observed (*C. coli*, 3.7% and *C. jejuni*, 4.4%) and it was similar to that reported in poultry isolates by Rozynek [58]. Resistant mutants develop slowly during antibiotic treatment. Subtherapeutic doses of tylosin given continuously in food exert a more significant influence on development of macrolide-resistant *Campylobacter* then therapeutic use of this antibiotic in chicken. Ladely et al. [63] found that long period of exposure to macrolides leads to macrolide resistance in *Campylobacter*. The studies of Hao et al. [64] compared the fitness of erythromycin-resistant and susceptible *Campylobacter*. The fitness reduction observed in resistant *Campylobacter* may cause lower frequency of macrolide resistance in clinical isolates. The continuous use of macrolides in food-producing animals at subtherapeutic concentrations is a major risk factor influencing the emergence of erythromycin resistance of *Campylobacte*r isolated from animals and environmental sources [63, 65, 66].

The most frequent resistance pattern observed in this study was the lack of susceptibility to a single agent, which was characteristic for all isolates. Double resistance to ciprofloxacin and tetracycline was detected most frequently both in children (28.6% *C. coli* and 36.9% *C.jejuni*), and in pet (33.3, 22.6%) isolates, and this percentage was higher than that reported by other authors [15, 67]. It should also be emphasised that one *C. jejuni* isolate from a fountain was simultaneously resistant to erythromycin, azithromycin, tetracycline and ciprofloxacin. A water sample was taken on a warm sunny day in May from the ornamental fountain located in the City Park near a children’s playground. Water was sprayed 1.2 m into the air and fell down into the fountain basin with a concrete bottom and a water surface area of ∼40 m^2^. The fountain had a closed system in which recirculating water could stagnate, whereas the total water replacement took place only once or twice during their exploitation. The fountain was not equipped with disinfecting or filtering devices. This shows the necessity of regular control not only of the design but also of operation of water facilities used for recreation.

Antimicrobials used in the rearing of food-producing animals cause strong selection of bacteria including pathogenic ones. Genotypes resistant to antimicrobial compounds can spread from farms into the environment. The natural reservoirs such as surface water, pets and wild birds are under threat of contamination with these bacteria.

## Conclusions

This is the first study on urban ornamental lakes and fountains as potential reservoirs of *Campylobacter* and one of few that provide data on the antimicrobial resistance of *Campylobacter* isolated from surface water. The results of our study show that fountains can be a direct source of children campylobacteriosis but also a source of cross-contamination of other environments with multidrug-resistant *Campylobacter.* Consequently, regular monitoring of water facilities used for recreation is necessary. This study suggests that surface water, poultry meat and pets can be potential sources of *Campylobacter* to children. High resistance among the isolates to some antimicrobials may lead to increasing numbers of difficult-to-treat campylobacteriosis in children. Therefore, constant monitoring of resistance is required in both children and environmental *Campylobacter* isolates. In order to improve the understanding of the complex epidemiology of *Campylobacter* infections further studies should be conducted on larger population and on various sampling groups, with the use of molecular techniques.
